# Likelihood of Bacterial Infection in Immunocompromised Patients Treated With IV Antibiotics for Possible Sepsis

**DOI:** 10.1097/CCE.0000000000001443

**Published:** 2026-07-15

**Authors:** Simran Gupta, Lucia Millham, Laura DelloStritto, Anna A. Agan, Cara McKenna, Chanu Rhee, Michael Klompas

**Affiliations:** 1 Department of Medicine, Brigham and Women’s Hospital, Boston, MA.; 2 Harvard Medical School, Boston, MA.; 3 Department of Population Medicine, Harvard Pilgrim Health Care Institute, Boston, MA.

**Keywords:** bacterial infection, immunocompromised host, infection, sepsis

## Abstract

**OBJECTIVES:**

Immunocompromised patients with possible sepsis are typically treated immediately with empiric antibiotics given their high risk for poor outcomes. Clinical presentations of sepsis, however, are protean and many nonbacterial infections and noninfectious conditions can present similarly. We assessed the likelihood of bacterial infections in retrospect in immunocompromised vs. nonimmunocompromised patients treated empirically for sepsis.

**DESIGN, SETTING AND PATIENTS:**

We identified all adults treated for possible sepsis (blood culture draw, lactate measurement, and IV antibiotic administration) within 6 hours of emergency department (ED) arrival at nine hospitals between 2022 and 2024 and randomly selected 350 hospitalizations for structured medical record review, oversampling for immunocompromised status using administrative codes. Reviews were used to confirm immunocompromised status per Centers for Disease Control and Prevention criteria and determine post hoc likelihoods of bacterial infection.

**INTERVENTIONS:**

None.

**MEASUREMENTS AND MAIN RESULTS:**

Of the 350 patients, 186 (53.1%) were confirmed as immunocompromised and 164 (46.9%) as nonimmunocompromised. Immunocompromised patients more commonly presented with fevers, chills or rigors (66.0% vs. 32.0%, *p* < 0.001) and ED clinicians more often documented concern for sepsis (74% vs. 62%, *p* = 0.01) compared with nonimmunocompromised patients. Definite or probable bacterial infection was identified in 92 of 186 (49.5%) immunocompromised patients vs. 97 of 164 (59.1%) nonimmunocompromised patients (*p* = 0.07). Among immunocompromised patients without definite or probable bacterial infection, common alternative etiologies included viral or fungal infection, febrile neutropenia without clinical evidence of bacterial infection, progression of malignancy, and medication toxicity, whereas chronic heart or lung disease exacerbations, hypovolemia, and hypervolemia predominated among nonimmunocompromised patients.

**CONCLUSIONS:**

Only about one-half of immunocompromised patients treated empirically for possible sepsis had definite or probable bacterial infection in retrospect, compared with approximately three-fifths in nonimmunocompromised patients. These findings underscore the complexity of sepsis diagnosis, particularly in immunocompromised patients, and highlight important differences in the conditions that mimic bacterial sepsis in immunocompromised vs. nonimmunocompromised hosts.

Diagnosing bacterial sepsis and knowing when to start empiric antibiotics in immunocompromised hosts can be challenging. Clinical signs may be blunted or atypical, nonbacterial pathogens such as viruses or fungi are common, and noninfectious mimics—such as malignancy-related syndromes, inflammatory disorders, and medication toxicities—are frequent (1). Clinicians recognize, however, that immunocompromised patients are uniquely vulnerable to poor outcomes and many consequently have low thresholds to start empiric broad-spectrum antibiotics.

Studies in unselected patients treated for possible sepsis document that a third or more have nonbacterial conditions (2, 3). The analogous proportion in immunocompromised patients is unclear. It may be lower given immunocompromised patients’ predilection to severe bacterial infections, or conversely, it may be higher given their greater risk for fungal and viral infections, and the broader array of possible sepsis mimics conferred by their underlying conditions and treatments.

Understanding how often immunocompromised patients treated for possible sepsis have confirmed bacterial infections is important to inform clinical decision-making, guidelines, and quality improvement initiatives seeking to balance the urgency of antibiotics for bonafide bacterial infections vs. minimizing antibiotic overuse. We therefore conducted detailed chart reviews to quantify the post hoc likelihood of bacterial infection in immunocompromised vs. nonimmunocompromised patients treated with IV antibiotics for possible sepsis in the emergency departments (EDs) of nine hospitals.

## METHODS

We performed a retrospective study of patients older than 18 years presenting between 2022 and 2024 to nine Mass General Brigham EDs (two academic and seven community hospitals). Possible sepsis was defined by blood culture and lactate measurements (regardless of result) and any IV antibiotic administered within six hours of ED arrival, identified using the hospitals’ centralized Enterprise Data Warehouse. We identified potentially immunocompromised patients using an *International Classification of Diseases*, Tenth Revision-based screening strategy adapted from the Food and Drug Administration Center for Biologics Evaluation and Research *Biologics Effectiveness and Safety Initiative* framework for identifying immunocompromised hosts, supplemented by clinical and treatment-related data to enrich for patients with clinically significant immunocompromise (**eMethods 1,** https://links.lww.com/CCX/B653) (4). The study was approved by the MGB institutional review board (Protocol Number 2018P000602) with a waiver of informed consent.

We randomly selected 350 patients, oversampled for potentially immunocompromised patients (*n* = 250, vs. 100 nonimmunocompromised) and conducted detailed medical record reviews to a) confirm immunocompromised status using Centers for Disease Control and Prevention (CDC) criteria (5), and b) determine the post hoc likelihood of bacterial infection. All available notes, vital signs, medications, laboratory and microbiology tests, radiology reports, and pathology reports were reviewed.

We classified the likelihood of bacterial infection as “definite,” “probable,” “possible but unlikely,” or “highly unlikely/definitely not” per prior studies (3, 6). “Definite” required objective microbiologic or pathologic confirmation; “probable” indicated a compatible syndrome responsive to antibiotics and no alternative diagnosis; “possible but unlikely” indicated infection could not be excluded but a noninfectious process was more likely; and “highly unlikely/definitely not” indicated a clear noninfectious etiology (additional details in **eMethods 2**, https://links.lww.com/CCX/B653).

The first 20 cases were independently reviewed by four physicians (S.G., L.M., C.R., M.K.) and discussed in person to adjudicate discrepancies and ensure a standardized process. The remaining cases were reviewed by two physicians (S.G., L.M.); if either was uncertain how to classify a case, it was discussed by all four reviewers to reach consensus (*N* = 16 cases).

Characteristics and likelihood of infection, as well as alternative diagnoses for possible but likely or highly unlikely/definitely no infection, were compared between immunocompromised and nonimmunocompromised cohorts. Two tailed *z*-tests were used to assess statistically significant differences in bacterial infection rates and other categorical variables. Analyses were conducted using SAS (Version 9.4; SAS Institute, Cary, NC). The study was approved by the Mass General Brigham institutional review board with a waiver of informed consent.

## RESULTS

There were 28,988 patients with possible sepsis during the study period including 12,265 potentially immunocompromised and 16,723 presumed nonimmunocompromised (per administrative codes). Among the 350 study patients, 186 (53.1%) were confirmed on medical record review to meet CDC criteria for immunocompromised status and 164 (46.9%) did not (immunocompromised conditions summarized in **eTable 1**, https://links.lww.com/CCX/B653).

### Patient Characteristics

Among the 350 reviewed cases, immunocompromised patients were slightly younger than nonimmunocompromised patients (mean age 63.2 vs. 65.8 yr), had more comorbidities (median Elixhauser comorbidity index 17 vs. 5), and higher inpatient mortality (7.5% vs. 3.7%). Median hospital length-of-stay (6 vs. 5 d) and ICU lengths-of-stay (2 d each) were similar (**Table 1**).

**TABLE 1.** Characteristics of Immunocompromised and Nonimmunocompromised Patients in the Study

Demographics and Characteristics	Immunocompromised (*n* = 186)	NonImmunocompromised (*n* = 164)
Demographics		
Age, yr, mean (sd)	63.2 (14.0)	65.8 (17.7)
Male sex, *n* (%)	92 (49.5)	101 (61.6)
White race, *n* (%)	143 (76.9)	125 (76.2)
Elixhauser comorbidities, *n* (%)		
Cancer	121 (65.1)	21 (12.8)
Diabetes	50 (26.9)	59 (36.0)
Chronic lung disease	42 (22.6)	51 (31.1)
Congestive heart failure	32 (17.2)	41 (25.0)
Renal failure	33 (17.7)	15 (9.1)
Presence of explicit infectious symptoms, *n* (%)	147 (79.0)	98 (59.8)
First antibiotic administered in the ED, *n* (%)		
Cefepime	102 (54.9)	45 (27.4)
Piperacillin–-tazobactam	13 (7.0)	30 (18.3)
Ceftriaxone	40 (21.5)	58 (35.4)
Meropenem	5 (2.7)	3 (1.8)
Vancomycin	14 (7.5)	11 (6.7)
Ampicillin–sulbactam	0 (0)	4 (2.4)
Levofloxacin	1 (0.5)	4 (2.4)
Median time from ED arrival to first IV antibiotic, hr, IQR	2.58 (2.13)	2.43 (2.26)
Duration of antibiotics, d, median (IQR)	5 (5)	4 (4)
ED discharge location, *n* (%)		
Ward	167 (89.8)	138 (84.1)
ICU	17 (9.1)	23 (14.0)
ED observation	1 (0.5)	3 (1.8)
Home	0 (0)	0 (0)
Positive blood culture, *n* (%)		
*Excluding contaminants*	25 (13.5)	15 (9.1)
*Staphylococcus aureus* (methicillin-resistant *S. aureus* and methicillin-sensitive *S. aureus*)	1 (4.0)	2 (13.3)
*Streptococcus* species	2 (8.0)	2 (13.3)
Coagulase-negative staphylococcus	3 (12.0)	1 (6.7)
Enterococcus species	4 (16.0)	3 (20.0)
*Escherichia coli*	6 (24.0)	2 (13.3)
*Klebsiella* species	4 (16.0)	4 (26.7)
*Pseudomonas aeruginosa*	5 (20.0)	0 (0.0)
*Enterobacter* species	1 (4.0)	0 (0.0)
Other	4 (16.0)	4 (26.7)
Positive non-blood culture/assay (bacterial), *n* (%)	71 (38.2)	82 (50.0)
Positive nonbacterial culture/assay, *n* (%)	43 (23.1)	18 (11.0)
Fungal, *n* (%)	9 (20.9)	1 (5.6)
Viral, *n* (%)	36 (83.7)	18 (100.0)
Parasitic, *n* (%)	2 (4.7)	0 (0.0)
Outcomes		
Hospital LOS, d, median (IQR)	6 (5)	5 (6.25)
ICU LOS, d, median (IQR)	2 (3)	2 (4)
Died during encounter, *n*, %	14 (7.5)	6 (3.7)

ED = emergency department, IQR = interquartile range, LOS = length of stay.

### Clinical Presentation

Immunocompromised patients were more likely than nonimmunocompromised patients to present with objective or subjective fevers, chills or rigors (66.0% vs. 32.0%, *p* < 0.001). Nonimmunocompromised patients were more likely to deny these symptoms (21.0% vs. 40.0%, *p* < 0.001). Mean maximum temperature within 24 hours of ED arrival was higher in immunocompromised vs. nonimmunocompromised patients (100.4°F vs. 99.9°F, *p* = 0.02) (additional details in **eTable2,** https://links.lww.com/CCX/B653). ED providers were more likely to document explicit concern for sepsis in immunocompromised vs. nonimmunocompromised patients (74.0% vs. 62.0%, *p* = 0.01) but similarly likely to document concern for infection (59.7% vs. 59.1%, *p* = 0.92) (Table 1).

### Microbiological Findings

Immunocompromised patients had fewer positive bacterial tests (culture, PCR, or antigen) compared with nonimmunocompromised patients (38.2% vs. 50.0%, *p* = 0.03). Blood cultures were positive in 32 of 186 (17.2%) immunocompromised patients vs. 27 of 164 (16.5%) nonimmunocompromised patients (*p* = 0.86); 25 of 32 (78.1%) were considered true pathogens in immunocompromised vs. 15 of 27 (55.6%) in nonimmunocompromised patients (*p* = 0.06). Immunocompromised patients were more likely to have positive nonbacterial pathogen tests (23.1% vs. 11%, *p* = 0.003), particularly respiratory viral swabs and 1,3-beta-d glucan assays.

### Post Hoc Likelihood of Bacterial Infection

**Figure 1.**
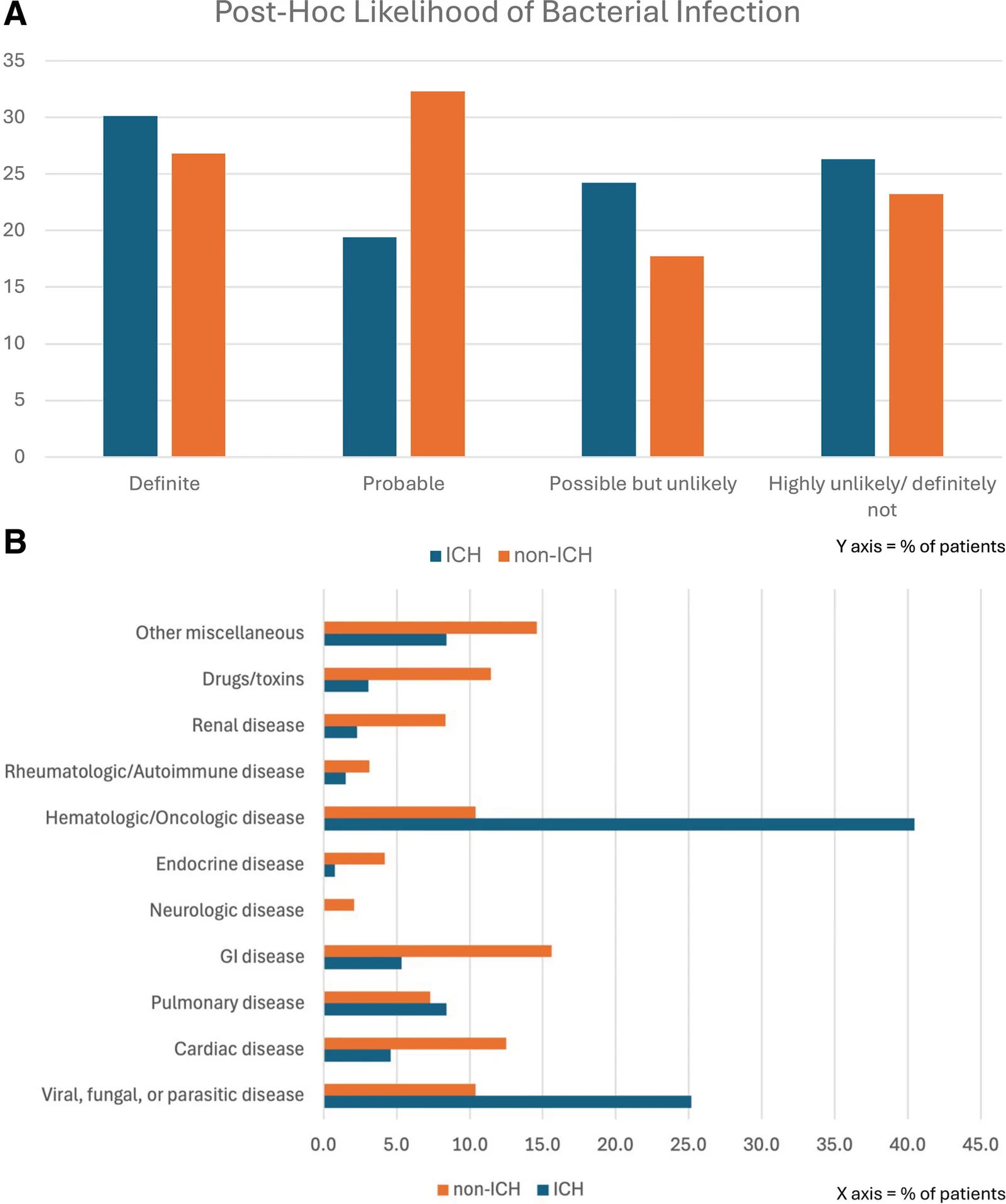
Post hoc likelihood of bacterial infection and alternative etiologies. **A**, Post hoc likelihood of bacterial infection stratified by immunocompromised and nonimmunocompromised status. **B**, Alternative etiologies for the presenting syndrome for patients who had possible but unlikely and highly unlikely/definitely no bacterial infection, stratified by immunocompromised and nonimmunocompromised status. GI = gastrointestinal, ICH = immunocompromised.

Definite bacterial infection was identified in 56 of 186 (30.1%) immunocompromised vs. 44 of 164 (26.8%) nonimmunocompromised patients (*p* = 0.50), probable infection in 36 of 186 (19.4%) vs. 53 of 164 (32.3%) (*p* = 0.005); possible but unlikely infection in 45 of 186 (24.2%) vs. 29 of 164 (17.7%) (*p* = 0.14); and highly unlikely/definitely not infected in 49 of 186 (26.3%) vs. 38 of 164 (23.2%) (*p* = 0.49) (**Fig. 1**). Overall, definite or probable bacterial infection was present in 49.5% of immunocompromised vs. 59.1% of nonimmunocompromised patients (*p* = 0.07).

Among immunocompromised patients with definite or probable bacterial infection, the most common sources were pulmonary (26.1%), urinary (23.9%), intra-abdominal (21.7%), primary bacteremia (13.0%) and skin and soft tissue (13.0%). In nonimmunocompromised patients, pulmonary (35.1%), skin and soft tissue (25.8%), urinary (20.6%), intra-abdominal (12.4%), and endovascular (3.1%) infections predominated. Aspiration events accounted for 6 of 92 (6.5%) definite or probable infections in immunocompromised cases vs. 17 of 97 (17.5%) definite or probable infections in nonimmunocompromised cases.

### Alternative Diagnoses

When bacterial infection was possible but unlikely or highly unlikely/definitely not present, the most common alternative diagnoses in immunocompromised patients were febrile neutropenia without clinical evidence of bacterial infection (39.6% vs. 0.0% in nonimmunocompromised), viral infection (29.7% vs. 12.0%), fungal infection (7.7% vs. 0.0%), progression or complication of known malignancy (9.9% vs. 3.3%), or toxicity from chemotherapy or immunosuppressive medications (8.8% vs. 0%). In nonimmunocompromised patients, volume overload or cardiac disease (19.6% vs. 6.6%), hypovolemia (8.7% vs. 5.5%), and chronic lung disease exacerbations (7.6% vs. 3.3%) predominated.

## DISCUSSION

Only about one-half of immunocompromised patients treated in the ED with IV antibiotics for possible sepsis had definite or probable bacterial infection in retrospect, compared with nearly 60% of nonimmunocompromised patients (*p* = 0.07). The most common sepsis-mimicking syndromes differed between groups: most immunocompromised patients had febrile neutropenia without a clear trigger, viral infection, fungal infection, complications of cancer, or medication toxicities, whereas nonimmunocompromised patients most often had exacerbations of chronic heart and lung disease, hypovolemia, or volume overload.

Our findings are consistent with prior studies suggesting that 20–40% of patients treated for possible sepsis are either uninfected or have nonbacterial infections, but suggest that this proportion may be even higher among immunocompromised patients (2, 7–9). Immunocompromised patients can manifest a wider array of bacterial sepsis mimics due to their underlying conditions, specialized treatments, and increased susceptibility to nonbacterial pathogens. They may also present with atypical symptoms of infection, increasing diagnostic uncertainty and prompting empiric antibiotic use, and the threshold to start empiric antibiotics may be lower in this population.

Our findings underscore the need to balance the urgency of treating true bacterial infections with the risks of antibiotic overtreatment, including selection for multi-drug-resistant organisms, *Clostridioides difficile* infection, gut microbiome disruption, antimicrobial adverse effects, and interference with noninfectious therapeutics (10). Clinicians remain constrained, however, by the low specificity of many clinical syndromes and the limited speed and accuracy of current diagnostics. Importantly, some patients may still require broad empiric therapy — not only with antibiotics, but also antivirals or antifungals. Indeed, in our study, immunocompromised patients demonstrated greater evidence of viral and fungal infections, as well as overall higher mortality. Thus, stewardship in this context requires not just reducing unnecessary antibiotic exposure, but also improving diagnostic precision to ensure appropriate empiric coverage across a wider range of potential pathogens.

Our study has limitations. First, it was conducted within a single healthcare system, potentially limiting generalizability to other practice environments. We did, however, include nine community and academic centers with diverse patient populations and practice patterns. Second, post-hoc determination of the likelihood of bacterial infection can be subjective. To mitigate this, we used a structured review process consistent with prior studies, a priori definitions for each infection likelihood category, overlapping review of an initial set of cases, and consensus discussions among four physicians for complex cases (2, 9). We also took a conservative approach to classifying aspiration events, classifying most as probable bacterial pneumonias despite the possibility that some may have been pneumonitis alone. Third, we did not assess the consequences of antibiotic overtreatment; however, this has been well established in the literature (9). Finally, initial identification of immunocompromised patients relied on administrative coding, though this was followed by manual chart reviews using CDC criteria to minimize misclassification.

In conclusion, only about one-half of immunocompromised patients treated for suspected sepsis in the ED had definite or probable bacterial infection in retrospect, compared with approximately three-fifths in nonimmunocompromised patients. These findings underscore the diagnostic challenges and risk of overtreatment for patients with possible sepsis, particularly in immunocompromised patients, and highlight the need for refined diagnostic strategies and antimicrobial stewardship in this vulnerable population.

## Supplementary Material
