# Likelihood of Bacterial Infection in Immunocompromised Patients Treated With IV Antibiotics for Possible Sepsis: Erratum

**DOI:** 10.1097/CCE.0000000000001485

**Published:** 2026-09-09

**Authors:** Simran Gupta, Lucia Millham, Laura DelloStritto, Anna A. Agan, Cara McKenna, Chanu Rhee, Michael Klompas

In the article published in Volume 8, Issue 7 of *Critical Care Explorations*, the expansion of ICH in the caption of Figure 1 was incorrect. The correct expansion is: immunocompromised. This error has now been corrected in both the online and PDF versions of the article.
