# Supplementary material for: Likelihood of Bacterial Infection in Immunocompromised Patients Treated With IV Antibiotics for Possible Sepsis
Source: Crit Care Explor. 2026 Jul 15;8(7):e1443. doi: 10.1097/CCE.0000000000001443 (PMC13375051; doi:10.1097/CCE.0000000000001443)
Supplement: Supplementary file 1 [file cc9-8-e1443-s001.pdf]

Supplemental Digital Content

Likelihood of Bacterial Infection in Immunocompromised Patients Treated with Intravenous Antibiotics for Possible Sepsis

eMethods 1. Definitions for Post-Hoc Likelihood of Bacterial Infection

eMethods 2. Identification and Sampling of Immunocompromised Patients

eTable 1. Immunocompromising Conditions Included in the Cohort Based on CDC Criteria

eTable 2: Mean Maximum Temperatures within 24h of ED Arrival in Immunocompromised and Non-Immunocompromised Cohorts Stratified by Post-Hoc Likelihood of Bacterial Infection

eMethods 1: Definitions for Post-Hoc Likelihood of Infection

1. *Definite bacterial infection* was defined as a compatible clinical syndrome plus pathologic diagnosis of infection, operative identification of pus, positive culture from a normally sterile site (not likely to be a contaminant), or a positive culture from a non-sterile site with supportive clinical signs or imaging consistent with infection at that site *(probability >=90% that bacterial infection was present and responsible for the patient's presentation).*
2. *Probable bacterial infection* was defined as not meeting criteria for definite but having a compatible clinical syndrome responsive to antibiotics with no clear alternative explanation for the patient's syndrome (or if multiple potential etiologies, bacterial infection thought most likely) *(probability 50-89%)*.
3. *Possible but unlikely bacterial infection* was defined as a clinical syndrome with a non-infectious primary diagnosis but some signs potentially consistent with infection such as a positive culture and receipt of antibiotics, but with a more likely alternative diagnosis identified *(probability 11-49%)*.
4. *Highly unlikely / Definitely no bacterial infection* was deemed present if there was a clear non-infectious diagnosis and no evidence of a concurrent bacterial process *(probability <=10%)*.

eMethods 2. Identification of Immunocompromised Patients and Sampling Strategy

We used a structured screening strategy to identify patients with potentially immunocompromising conditions based on ICD-10 discharge diagnosis codes present during either 1) the index hospitalization or 2) a hospitalization with discharge date within the 6 months prior to the index hospitalization admission date.

This approach was adapted from FDA Center for Biologics Evaluation and Research (CBER) algorithm for immunocompromised hosts (ICH) (https://bestinitiative.org/wp-content/uploads/2022/05/Immunocompromised_Algorithm_Final_Report_2021.pdf) ICD-10 screening categories adapted from the FDA CBER framework included:

- HIV
- Hematologic malignancy
- Immune deficiency – treatment independent
- Immune deficiency – treatment dependent
- Solid malignancy
- Transplant and related conditions
- Rheumatologic/inflammatory conditions
- Steroid use

“Dialysis” and “intermediate” conditions included in the FDA CBER algorithm were excluded from our screening approach because of limited relevance to infection-related immunocompromise.

Because our initial electronic screening relied primarily on diagnosis codes without comprehensive outpatient medication data, we also identified a subgroup of patients highly likely to have clinically significant immunocompromise (“severe ICH”) in order to enrich the screened sample for true immunocompromised hosts. Specifically, we focused on high-risk diagnostic categories supplemented by clinical and treatment-related data such as neutropenia and receipt of immunosuppressive therapies as below.

Severe ICH was defined as any of the following:

a. ICD-10 codes for hematologic or solid malignancy with at least one absolute neutrophilc count (ANC) value < 1000 cells/μL (or if no ANC available, use a WBC < 1.0 cells/μL) within 24 hours of ED arrival
b. Solid or Bone Marrow Transplant with either of the following:
    i. At least 1 ANC value <1000 cells/μL within 24 hours of ED arrival OR
    ii. Receipt of ≥1 of the following immunosuppressive medications anytime during hospitalization: (tacrolimus, sirolimus, methylprednisolone, prednisone, cyclosporine, mycophenolate, azathioprine, everolimus, methotrexate).

c. Outpatient receipt of one of the following anti-CD20 antibodies within 90 days prior to the index hospitalization admission date: (rituximab, ocrelizumab, obinutuzumab, ofatumumab, ibritumomab, tositumomab, ublituximab).

Mild-Moderate ICH was defined as any patient with discharge diagnosis codes for potentially immunocompromising conditions in the FDA CBER algorithm who did not meet criteria for severe ICH.

We randomly selected 150 severe-ICH and 100 mild-moderate ICH patients by FDA CBER criteria for medical record review and confirmation of ICH status by CDC criteria developed for defining risk in the context of COVID-19 (https://archive.cdc.gov/www_cdc_gov/coronavirus/2019-ncov/need-extra-precautions/people-who-are-immunocompromised.html). Only patients with ICH status confirmed on medical record review were included as ICH in analyses comparing likelihood of bacterial infection between ICH and non-ICH groups.

The positive predictive value of administrative coding data compared to the manually adjudicated CDC criteria for ICH status was 97.3% (146/150; 95% CI 93.7,99.0%) for severe-ICH and 40% (40/100; 95% CI (30.9, 49.7%) for mild to moderate ICH. All 100 electronically identified non-ICH patients were confirmed as non-immunocompromised per CDC criteria on manual review [100%; 95% CI; 96.3, 100%).

eTable 1: Immunocompromising Conditions Included in the Cohort Based on CDC Criteria

**Immunocompromising Conditions**, n (%)	**Immunocompromised (n=186)**
Solid or hematologic malignancy on treatment	93 (26.6)
Hematologic malignancy	65 (18.6)
SOT on immunosuppression	68 (19.4)
CAR-T or HCT in last 2 years or on immunosuppression	18 (5.1)
Moderate or severe primary immunodeficiency	1 (0.3)
Advanced or untreated HIV	1 (0.3)
Active treatment with highly immunosuppressive agents [+]	163 (46.6)

[+] Highly immunosuppressive agents include: high-dose corticosteroids (ie 20 or more mg of prednisone or equivalent per day when administered for 2 or more weeks), alkylating agents, antimetabolites, transplant-related immunosuppressive drugs, cancer therapeutic agents classified as severely immunosuppressive, tumor necrosis factor (TNF) blockers, and other biologic agents that are immunosuppressive or immunomodulatory

eTable 2: Mean Maximum Temperatures within 24h of ED Arrival in Immunocompromised and Non-Immunocompromised Cohorts Stratified by Post-Hoc Likelihood of Bacterial Infection

Post-Hoc Likelihood of Infection	Immunocompromised (n=186)	Non-immunocompromised (n=164)
Definite	100.4°F	100.8°F
Probable	100.3°F	99.9°F
Possible but Unlikely	100.5°F	99.9°F
Highly Unlikely/Definitely Not	100.4°F	99.1°F
